# Finite Element Analysis of Electrospun Nanofibrous Mats under Biaxial Tension

**DOI:** 10.3390/nano8050348

**Published:** 2018-05-19

**Authors:** Yunlei Yin, Jie Xiong

**Affiliations:** 1 School of Materials and Textiles, Zhejiang Sci-Tech University, Hangzhou 310018, China; Yinyunlei66@126.com; 2 Key Laboratory of Advanced Textile Materials and Manufacturing Technology of Ministry of Education, Zhejiang Sci-Tech University, Hangzhou 310018, China

**Keywords:** electrospun, biaxial tension, mechanical behavior, finite element analysis

## Abstract

Due to the non-uniform material properties of electrospun nanofibrous mats and the non-linear characteristics of single fibers, establishing a numerical model that can fully explain these features and correctly describe their properties is difficult. Based on the microstructure of electrospun nanofibrous mats, two macroscopic continuum finite element (FE) models with a uniform or oriented nanofiber distribution were established to describe the mechanical behavior of nanofibrous mats under biaxial tension. The FE models were verified by biaxial tension experiments on silk fibroin/polycaprolactone nanofibrous mats. The developed FE models expressed the mechanical behaviors of the mats under biaxial tension well. These models can help clarify the structure–property relationship of electrospun nanofibrous mats and guide the design of materials for engineering applications.

## 1. Introduction

Electrospun nanofibrous mats have a special fiber structure composed of continuous layers of numerous and long micro/nanofibers. The fibers constituting the nanofibrous mats are produced by high-voltage electrospinning, which uses high-voltage static electricity to produce a charged-polymer jet to stretch and thin the fibers in the electrostatic field. After solvent evaporation and jet solidification, a web is formed on the device. When the micro/nanoscale monofilaments are stacked and merge, the final material shows unique performance. For example, their volume ratio is increased, and the porosity is high. The fiber surface created by the combination of the nanostructure and the connected porous structure is highly suitable for cell adsorption and multiplication. These special structures are particularly beneficial for the field of biological tissue engineering [1,2,3,4,5,6]. Therefore, biocompatible nanofibrous mats with three-dimensional structures are a hot research topic in tissue engineering. In addition, the morphological structure of electrospun nanofibrous mats makes them suitable for applications in air or liquid filters [7,8], reinforcing materials [9,10] and sensors [11,12], among others. 

The mechanical properties of electrospun nanofibrous mats largely guide their applications. Their mechanical functionality is the most direct and basic behavior in determining their ideal application. As a tissue engineering scaffold in cell culture and tissue formation, electrospun mats must provide mechanical performance support at both the microscopic and macroscopic level [13]. Studies [14] have shown that the mechanical properties of scaffold materials are key to the effectiveness of scaffolds in tissue engineering and regenerative medicine. From a microscopic perspective, nanofibers in the scaffolds must have sufficient strength and toughness to support cell attachment, growth, and migration, as well as deposition of the extracellular matrix. At the same time, from a macroscopic perspective, nanofibrous mat scaffolds must have mechanical properties in line with the substitute tissues. However, investigations have focused on manipulation of the chemical content and physical properties of the electrospun nanofibrous materials to produce materials with good functionalities for various purposes [15,16,17]. The required mechanical properties of the materials are often ignored or simply discussed, and few modeling analyses of the mechanical response has been reported. In fact, under the complicated loading conditions, the development of a high-efficiency simulation based on the tensile constitutive relation of electrospun nanofibrous mats is an urgent problem to be solved for engineering applications. In addition, simple and effective models can simulate the mechanical properties of the scaffold materials under different loading conditions. Meanwhile, complicated and arduous experimental work can be avoided, and the design and prediction of the mechanical properties of the material can be made easier.

The mechanical properties of nanofibrous mats are determined by their microstructure and nanofibrous properties. Since Backer and Petterson’s pioneering work [18], researchers have made considerable effort to develop methods to describe these unique materials. Dupaix et al. [19] modified the 8-chain rubber elasticity model of Arruda et al. [20] to capture the mechanical behavior of electrospun polycaprolactone. Silberstein et al. [21] proposed a model to predict the macroscopic mechanical behavior of electrospun polyamide nanofibers. This model consists of a multi-layer triangular network and uses a homogenization technique to predict a response to monotonic and cyclic loading. These models did not predict the localization of damage or changes in the microstructure of the material. To overcome these shortcomings, microstructure-based models employing the direct introduction of individual fibers according to their orientation distribution were developed [22,23,24,25,26,27]. Ridruejo et al. [28] proposed a similar model requiring the introduction of a non-continuous microstructure but that does not use the actual fiber orientation in fabrics. In addition, Isaksson et al. [29] and Wilbrink et al. [30] proposed analysis models focusing on crack propagation and cohesion failure in fiber webs. Although these models based on different viewpoints are useful for analyzing mechanical behaviors and mechanisms of nonwoven fiber web deformation and failure, they only solve some problems, and the mesostructure of the actual material can be shown only by introducing fibers and the constituent fiber properties. However, they cannot predict the evolution of the fabric from deformation, damage to failure, i.e., the gradual destruction process. Farukh et al. [27] used a parameterized modeling technique based on a specially developed user subroutine for modeling the actual microstructure of a thermally bonded nonwoven fiber network in a finite element (FE) environment. They introduced the fiber directly into the model according to the fiber orientation distribution in the fabric fiber web. This enabled the model to obtain the random anisotropy of the web. Meanwhile, changes in the elastic-plastic mechanical properties of the constituent fibers and the single fiber failure criterion were also introduced into the model. They used this criterion to control the occurrence and evolution of fiber damage in the model. Through this model, they obtained the mechanism of deformation and failure progression of thermally bonded fabrics under uniaxial tension. Yin et al. [31] established a quantitative relationship based on the tensile properties of single nanofibers and nanofibrous mats in consideration of the experimental difficulty of measuring single fibers. Meanwhile, the effects of the fiber orientation distribution, fibrous curvature, fibrous mat porosity and length-wide ratio on the tensile properties of the nanofibrous mats were considered to build a uniaxial tensile stress-strain constitutive relation, and the model was verified by experimental data. However, when applied as a scaffold material, the electrospun nanofibrous mats are typically under planar force. Uniaxial stress analysis cannot fully reflect the actual stress state of the material. Therefore, double-axis mechanical tensile analysis must be conducted on the electrospun nanofibrous mats.

The purpose of this study is to analyze the mechanical response of electrospun nanofibrous mats under biaxial tension and to develop a simple and effective FE model. Application of the developed model can help clarify the relationship between the structure and performance of the fiber mats and provide guidance for designing materials, for example, to meet the needs of engineering applications. Electrospun nanofibrous mats with their actual microstructure were modeled here in an FE environment using a parametric modeling technique. The nature of the mats was captured by introducing fibers directly into the model according to their orientation distribution in the mats. In the two cases of uniform nanofiber distribution and oriented nanofiber distribution, the tensile behavior of the silk fibroin (SF)/polycaprolactone (PCL) nanofibrous mats under biaxial loading was analyzed. The FE models were assessed by experimental data.

## 2. Materials and Methods

### 2.1. Materials

SF with excellent mechanical strength and a high concentration of basic amino acids has been widely used as a bioactive dispersion phase for biological compound systems in tissue regeneration engineering. However, regenerated SF nanofibers obtained by electrostatic spinning mainly have a α-helix structure, which is an amorphous structure with poor mechanical properties, making it difficult to meet the requirements of a cell tissue engineering scaffold [32,33,34,35]. Blended spinning of SF with PCL, a biocompatible and biodegradable polymer material, can improve the mechanical properties of the fibers through synergistic effects of the polymer blend system without affecting the biological properties of SF [36,37].

The method for the extraction of regenerated SF described by Yin et al. [38] was followed using mulberry silk (Zhejiang Zhengqiang Textile Co., Ltd., Hangzhou, Zhejiang, China). PCL with a molecular weight of 8 × 10^4^ g mol^−1^ was obtained from Guanghua Weiye Co., Ltd., Shenzhen, China. 

### 2.2. Test Methods

A 6 wt % solution of the two SF and PCL polymers (3:2 mass ratio) were dissolved in 1,1,1,3,3,3-hexaflouro-2-propanol (Yancheng Dongyang Biological Products Co., Ltd., Yancheng, China). The electrostatic spinning parameters were as follows: voltage 15 kV, spinning flow rate 1.2 mL∙h^−1^, receiving distance 12 cm, drum rotation speed 11.88 m∙s^−1^, environment temperature 25 ± 2 °C, and relative humidity 35 ± 5%. 

The morphology of the SF/PCL composite nanofibrous mats was examined using a Vltra55 field-emission scanning electron microscope (FE-SEM; Carl Zeiss SMT Pte Ltd., Oberkochen, Germany) operating at 5 kV with a 16 mm working distance. Image-Pro Plus image analysis software (ICube, Crofton, MD, USA) was used to measure the diameter of the nanofibers. One hundred fibers were analyzed. The fiber orientation distribution was obtained by the DHU-11 nanofiber orientation image analysis system (Shanghai Beiang Scientific Instruments Co., Ltd., Donghua University, Shanghai, China). 

The porosity was determined by comparing the ratio of the measured mass of the specimen with the mass of a fully dense specimen of the same size by measuring the thickness, width, and length of the specimen. This method has been shown to provide results similar to a mercury porosimeter, as detailed by Rutledge et al. [39,40].

(1) $$P=\frac{M_{1}-M_{2}}{M_{1}}\;M_{1}=\left(L\times W\times T\right)\times \rho$$

where *P* is the porosity, *M_1_* is the mass of a fully compacted specimen of the same size as *M*_2_, *M*_2_ is the measurement of the sample quality, *L* is the length of the mat, *W* is the width of the mat, *T* is the thickness of the mat, and ρ is the density of the mat.

The tensile strength and the material strain against the tensile strength of the mats were determined according to the ISO 527-1 [41] and ISO 527-3 [42] standard test methods. The mats were mounted on a 50 mm × 50 mm biaxial tensile stage in a KSM-BX5450ST biaxial tensile test apparatus (Kato-Tech Company, Kyoto, Japan). The specimens were stretched at a constant engineering strain rate of 0.01 s^−1^.

## 3. Experimental Results

### 3.1. Characterization of the Mats

Electrospun nanofibrous mats should be collected in an orderly fashion for engineering applications to improve certain properties of the nanofibrous mats or to simulate the structural characteristics of the corresponding parts. In tissue engineering, oriented nanofibrous mats can substitute natural extracellular matrix (ECM) fibers to provide the appropriate mechanical strength and cell attachment sites and to regulate cell behaviors by altering the apparent topography. Therefore, orientation plays an important role in promoting cell growth and guiding tissue regeneration [43]. To obtain an oriented array of SF/PCL nanofibrous mats, a high-speed rotating roller was used to receive the nanofibers. 

**Figure 1:**
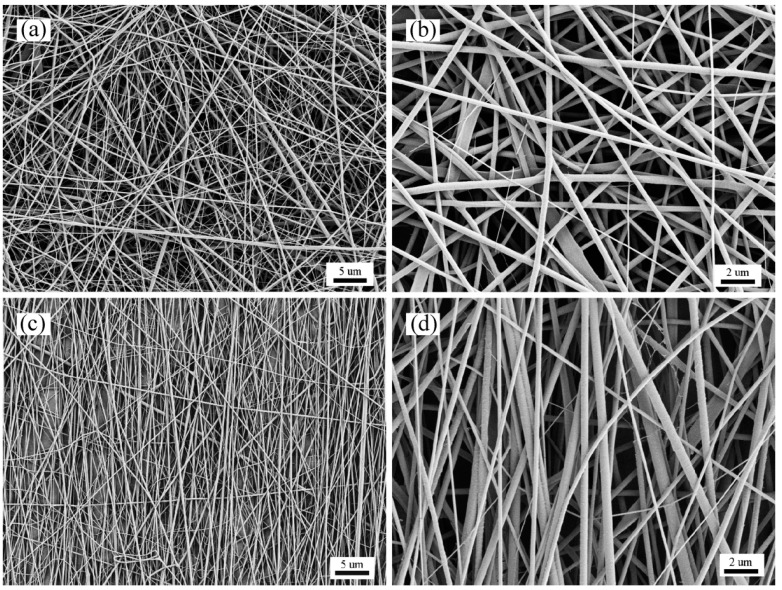
FE-SEM images of electrospun silk fibroin (SF)/polycaprolactone (PCL) nanofibrous mats. Here, (**a**) and (**b**) are nanofibrous mats produced under a roller speed of 0 m·s^−1^; (**c**) and (**d**) are nanofibrous mats produced under a roller speed of 11.88 m·s^−1^; (**a**) and (**c**), 2000× magnification (scale bar = 5 microns); (**b**) and (**d**), 5000× magnification (scale bar = 2 microns).

Thermal FE-SEM images of the SF/PCL nanofibrous mats are shown in Figure 1. The nanofibers were rod-shaped, and the surfaces of the fibers were smooth without beads. Furthermore, there was no bonding at the intersection of the fibers. When the rotation speed of the receiving roller was 0 m∙s^−1^, the average diameter of the nanofibers was 272 nm, and the standard deviation was 80 nm. At a receiving roller speed of 11.88 m∙s^−1^, the average diameter of the fibers was 260 nm with a standard deviation of 55 nm. With an increase in the rotation speed of the roller, the diameter of the fibers slightly decreased, but the overall change was small, approximately 272 nm. Therefore, increasing the rotation speed of the roller has little effect on the diameter of the SF/PCL nanofibers. According to the analysis, due to the optimization of the electrospinning technique, the nanofibrous are finer at a receiver rotation speed of 0 m·s^−1^. However, the fiber collection time from roller contact is very short, and the fiber diameter is too thin, and so the roller speed does not have an obvious effect on the fiber diameter.

**Figure 2:**
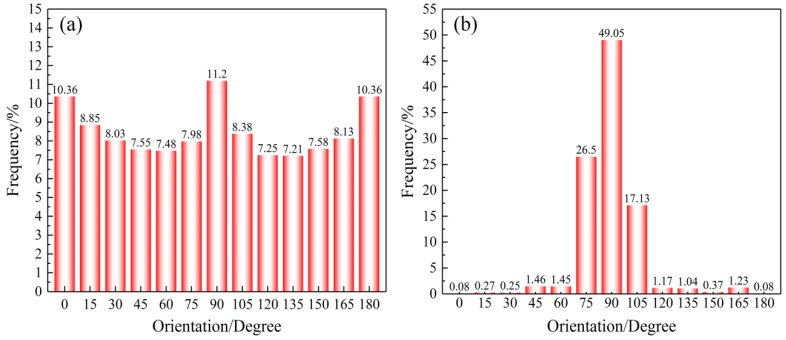
Fiber orientation distribution in the SF/PCL nanofibrous mats produced at (**a**) rotation speed=0 m∙s^−1^ and (**b**) rotation speed=11.88 m∙s^−1^.

The orientation distribution of the nanofibers was analyzed by the nanofibrous web orientation image analysis system. According to Figure 2, the fibers in the SF/PCL nanofibrous mats were approximately evenly distributed when the roller rotation speed was 0 m∙s^−1^. When the roller rotation speed was 11.88 m∙s^−1^, the fibers were mainly distributed at an angle of 90°, which was in line with the axis of the rotating roller (Y direction). The distribution of nanofibers in the direction perpendicular to the roller rotation direction (X direction) is extremely small, with a distribution rate of almost zero. Increasing the rotation speed of the roller can change the orientation distribution of the nanofibers and is an effective method to prepare oriented nanofibrous mats.

### 3.2. Mechanical Behavior

**Figure 3:**
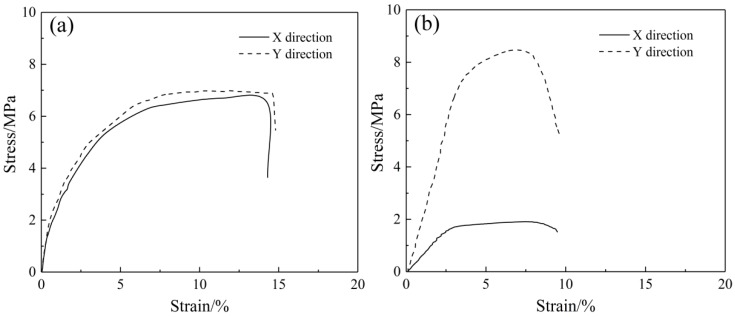
Biaxial stress-strain curves of the SF/PCL nanofibrous mats. (**a**) Uniform fiber distribution (rotation speed 0 m·s^−1^); (**b**) oriented fiber distribution (rotation speed 11.88 m·s^−1^).

The square tensile mats were fixed in a KSM-BX5450ST biaxial tensile test apparatus (Kato-Tech Company, Kyoto, Japan). The biaxial mechanical tensile curves of the SF/PCL nanofibrous mats at a biaxial tensile rate of 1:1 are shown in Figure 3. In Figure 3a, the nanofibrous mats exhibit similar stress-strain changes in both stretching directions. The mats show isotropic mechanical tensile properties. Figure 3b shows that with increasing rotation speed of the roller, the mechanical tensile properties of the nanofibrous mats in the X direction and the Y direction became significantly different, showing remarkable mechanical anisotropy. Meanwhile, the fracture strain of the SF/PCL nanofibrous mats was significantly reduced, with a decrease of approximately 7.8% at a rotational speed of 11.88 m∙s^−1^. This was because the destruction of the nanofibrous mats during tension under a plane force was determined by the strain in the minimum direction. At the same time, because numerous nanofibers are arranged along the tensile direction, the nanofibers were forced to extend until fracture during tension analysis. The fibers did not experience bending or straightening. Therefore, the nanofibrous membrane has a low fracture strain.

## 4. Constitutive Relationship

**Figure 4:**
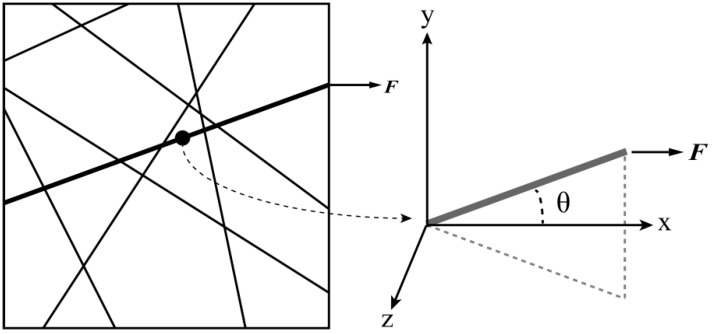
Configuration of the nanofibrous mats.

By referring to Figure 4, the following assumptions can be made before the derivation of the constitutive relationship:

1. Electrospun nanofibrous mats are assumed to be homogeneous and continuous on the macroscopic scale.
2. Single nanofibers are assumed to be incompressible and deform following the deformation of the mat.
3. Because electrospun nanofibrous mats are made by stacking many fiber layers with similar properties from one layer to the other, the deformation characteristics of the entire nanofibrous mat can be described by considering only a single layer. The material is transversely isotropic.

### 4.1. Uniaxial Tension

Referring to the research work of Yin et al. [31], the elastic modulus $E_{m}^{e}$ of the mats can be expressed as follows, where the fiber distribution density function is f(θ):

(2) $$E_{m}^{e}=E_{0}\left(1-P\right)SR\frac{\int_{0}^{\pi }f\left(\theta \right)cos^{4}\theta d\theta }{\int_{0}^{\pi }d\theta }$$

where $E_{0}$ is the elastic modulus of a single fiber, 1 − *P* is the volume fraction of the fibers, and *SR* is the stiffness ratio of the bent fiber to the straight fiber, and θ is the angle between the fiber and the stretching direction.

The modulus of the fibrous mats can be expressed as follows:

(3) $$E_{m}=\{\begin{array}{cc} E_{m}^{e}, & \varepsilon_{m}\leq \varepsilon_{m}^{y}\\ E_{m}^{'}, & \varepsilon_{m}>\varepsilon_{m}^{y} \end{array}$$

where $E_{m}^{'}$ is the strengthening modulus of the mat, $\varepsilon_{m}$ is the strain of the mat, and $\varepsilon_{m}^{y}$ is the yield strain of the mat.

As the tensile stress of the mat increases, the zero-angle fibers will first enter the plastic phase, followed by the $\theta_{1}$-angle fibers and finally the $\frac{\pi }{2}$-angle fibers. When the $\theta_{1}$-angle fibers enter the plastic phase, the stiffness of the mat can be separated into two parts. Fibers with angles greater than $\theta_{1}$ are still in the elastic phase, while fibers with angles less than $\theta_{1}$ are in the plastic phase.

(4) $$\frac{E_{m}^{'}}{E_{m}^{e}}=\frac{\int_{\theta_{1}}^{\frac{\pi }{2}}cos^{4}\theta d\;\theta +p\int_{0}^{\theta_{1}}cos^{4}\theta d\;\theta }{\int_{0}^{\frac{\pi }{2}}cos^{4}\theta d\;\theta }$$

For the $\theta_{1}$-angle fibers, the strain of the fibers in the mat can be written as

(5) $$cos^{2}\theta_{1}=\frac{\varepsilon_{m}^{y}}{\varepsilon_{m}}$$

Since we already know the elastic modulus $E_{0}$ of a single fiber and the volume fraction 1−P of the fiber web, we can calculate the initial elastic modulus $E_{m}^{e}$ of the fiber web using Equation (2). When the mats enter the plastic tension stage, as described in Equation (3), the total stress of the mats can be determined from the stress of the elastic section plus the stress of the plastic section, and the stress of the plastic section is calculated by the following steps: 1. $\theta_{1}$ is determined by the yield stress and strain of the fiber web and applied to Equation (5); 2. the value of $\theta_{1}$ is applied to Equation (4), where the proportional coefficient p is determined by the strengthening modulus and initial elastic modulus of the mat, $p=\frac{E_{m}^{'}}{E_{m}^{e}}$ and the area under the plastic stress-strain curve can be calculated following the change in $\theta_{1}$; and 3. The segmented area is summed to obtain the plastic stress. The elastic stress can be determined from the simple stress-strain relationship $\sigma =E_{m}^{e}\varepsilon$ to obtain the theoretical tensile stress-strain curve of the mats.

### 4.2. Biaxial Tension 

The constitutive relation of biaxial tension can be built through a similar uniaxial tensile constitutive relationship. For the web model distributed in the plane direction, the deformation gradient of 2D tension is:

(6) $$F=\frac{\partial x}{\partial X}=\left[\begin{array}{cc} F_{11} & F_{12}\\ F_{21} & F_{22} \end{array}\right]=\left[\begin{array}{cc} \lambda_{1} & 0\\ 0 & \lambda_{2} \end{array}\right]$$

where *X* is the position of a material point before deformation and *x* is the position of a material point after deformation. Assuming no shear deformation occurs during the application of tension, $F_{21}=F_{12}=0$. The above formula can be simplified to only the right two extension ratio parameters, $\lambda_{1}$ and $\lambda_{2}$:

(7) $$\lambda =\frac{r}{r_{0}}=\sqrt[{2}]{{\lambda_{1}}^{2}sin^{2}\theta +{\lambda_{1}}^{2}cos^{2}\theta }$$

where $r_{0}$ is the initial length of the fiber and r is the length after fiber tension.

The relationship between fiber displacement and the tension direction is:

(8) $$\delta =\sqrt{{\left({∆}_{1}+r_{0}sin\theta \right)}^{2}+{\left({∆}_{2}+r_{0}cos\theta \right)}^{2}}-r_{0}$$

where δ is the fiber direction displacement and ${∆}_{1}$ and ${∆}_{2}$ are displacements in two directions, which tend to be zero.

(9) $$\frac{\partial \delta }{\partial {∆}_{1}}=sin\theta {∆}_{1}$$

(10) $$\frac{\partial \delta }{\partial {∆}_{2}}=cos\theta {∆}_{2}$$

Assuming no shear action, the biaxial tension is not coupled and thus independent of each other, i.e., :

(11) $$\left[\begin{array}{cc} \sigma_{1} & 0\\ 0 & \sigma_{2} \end{array}\right]=\left[\begin{array}{cc} E_{1} & 0\\ 0 & E_{2} \end{array}\right]\left[\begin{array}{cc} \varepsilon_{1} & 0\\ 0 & \varepsilon_{2} \end{array}\right]$$

If the fibers are evenly distributed in all directions, the uniaxial tension constitutive relation can be used instead of the two-direction tensile constitutive relation. In fact, the modulus of the biaxial tension is slightly higher than that of uniaxial tension because of the limited aspect ratio of the specimen. Some fibers are not clamped under uniaxial tension, while fiber clamping can be achieved under biaxial tension.

## 5. Results and Discussion

### 5.1. Uniform Nanofiber Distribution

#### 5.1.1. Finite Element Modeling

Abaqus 11.3 (Providence, RI, USA) software was used for computational analysis, using the units of μm for length, μN for force, and MPa for stress. The diameter of the SF/PCL fiber was set to 0.272 μm, and the cell length was set to 1 μm. The material is assumed to be isotropic. The average elastic modulus of the fiber tensile curve was set to 1717 MPa. Poisson’s ratio was set to 0.3. The fiber was assumed to be a solid cylindrical structure. In addition, the porosity of the nanofibrous web was set to 75%. One thousand finite element model units were randomly distributed in the 100 μm × 100 μm grid, which was generated by the MATLAB R2012a program (Natick, MA, USA).

(12) 1−P=N×π×(D/2)2/10000/T

where *N* is the number of units and *D* is the fiber diameter.

**Figure 5:**
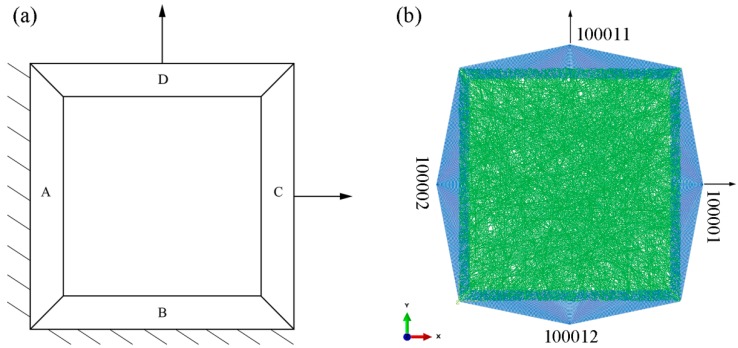
Boundary condition setting of biaxial tension. (**a**) schematic diagram; (**b**) loading mode.

A schematic drawing of the nanofibrous mat with imposed constraints and loads in the biaxial tension simulation is shown in Figure 5a. The 5 μm-long boundary of the square fiber mesh model is divided into four areas. The tension at C and D describe that in the X and Y direction, respectively. A forced displacement load of 9 μm is applied in the X direction and the Y direction. The degree of freedom in the Z direction is restricted. The symmetric boundary constraint is imposed on A and B in the X and Y direction, respectively. A constraint is imposed on A in the X and Z directions and on B in the Y and Z directions.

For conveniently imposing constraints and displacement loads in each area using the Abaqus 11.3 software and to facilitate the load output of each load after calculation, four nodes were established in the four areas, A, B, C, and D, as shown in Figure 5b. The four nodes are numbered as 10002, 100012, 100001, and 100011. Each node is bound to all the model nodes in the corresponding area using the TIE unit in the Abaqus 11.3 software. Constraints and displacements are applied only to the four nodes. The displacement and stress of two nodes at the right and top sides are the displacement and tension along the X axis and Y axis, respectively. Thus, the biaxial tensile stress-strain curves of the fibrous network can be obtained. The analysis is set to have 20 steps using the static/general solution.

#### 5.1.2. Biaxial Tensile Analysis

**Figure 6:**
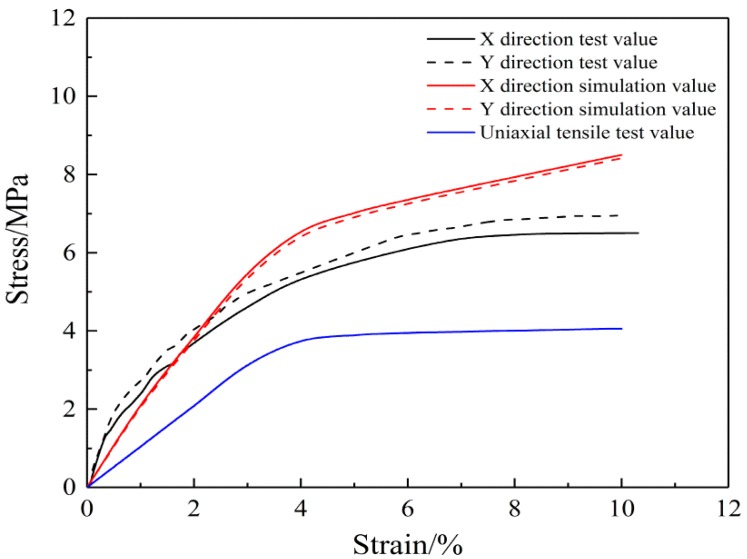
Biaxial tension of the SF/PCL nanofibrous mats (Uniaxial test data reproduced from reference [31], with permission from MDPI, 2018).

Figure 6 shows that when the nanofibers are evenly distributed, the stress-strain curve for the mats under the biaxial tension shows a similar trend as the experimental curve. In addition, the numerical values between the two directions of the mats are close. However, the modulus is higher than during uniaxial tension because many fibers that do not function during uniaxial tension, for example, fibers with two loading ends connected, also play a role. This leads to the SF/PCL nanofibrous mats having a larger modulus during biaxial tension than during uniaxial tension.

**Figure 7:**
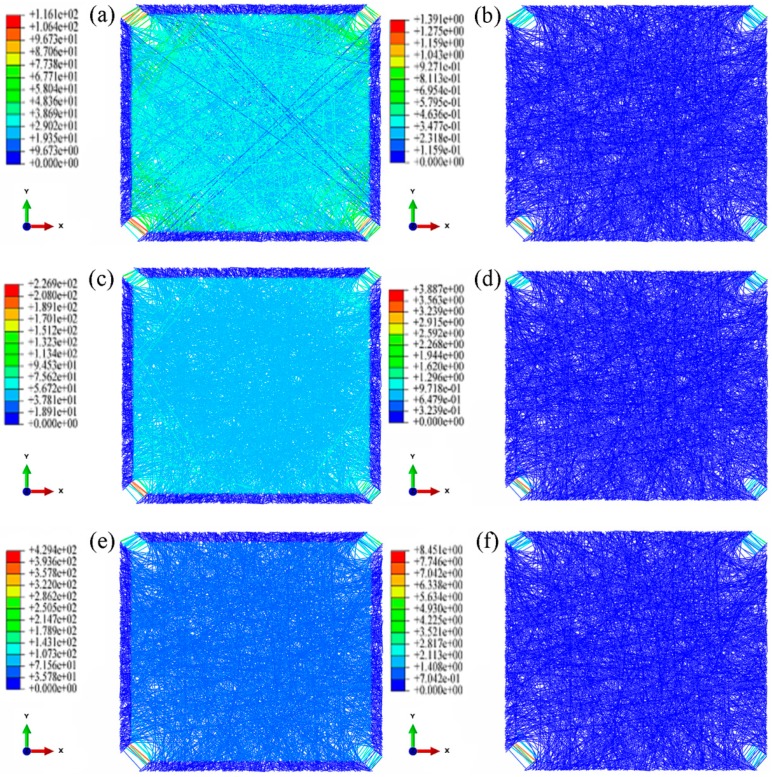
Biaxial tension cloud charts of uniform nanofibrous mats (aspect ratio 1:1, porosity 0.75). 1. strain = 2%, (**a**) stress (**b**) strain; 2. strain = 5%, (**c**) stress (**d**) strain; 3. strain = 10%, (**e**) stress (**f**) strain.

Figure 7 shows that, under biaxial loading, the fibrous mats are first torn in the four corners with a rapid increase in fiber stress and then enter into the yield phase (Figure 7a). With further loading, the number of fibers in the yield phase gradually increases, which then diffuse inside the nanofibrous mats (Figure 7c). Finally, under continuous loading, almost all fibers entered the yield phase (Figure 7e). This phenomenon is reflected in the stress-strain curve, which shows that with an increase in biaxial loading and tearing from the four corners inward, the fiber gradually enter the yield phase, while the slope of the stress-strain curve decreases continuously. This is similar to the trend of a parabola.

At strains of 0.03 to 0.05, the number of fibers entering the yield phase inside the fibrous mats rapidly increases, and the fibers gradually spread throughout the fibrous mats. Subsequently, when the strain of the fibrous mats reached 0.05, only a few fibers approaching 45° gradually entered the yield phase. Therefore, the stress-strain curve also significantly reduced in the strain range of 0.03 to 0.05, and the stress-strain curve could be divided into two sections.

#### 5.1.3. Stochastic Effect Analysis

Because a randomly generated fibrous network is used as the model in the simulation study, we also established two fibrous network models with the same parameters to research the effect of randomness. The aspect ratio and the porosity of the nanofibrous mats remained unchanged, and Model 2 and Model 3 were randomly generated.

**Figure 8:**
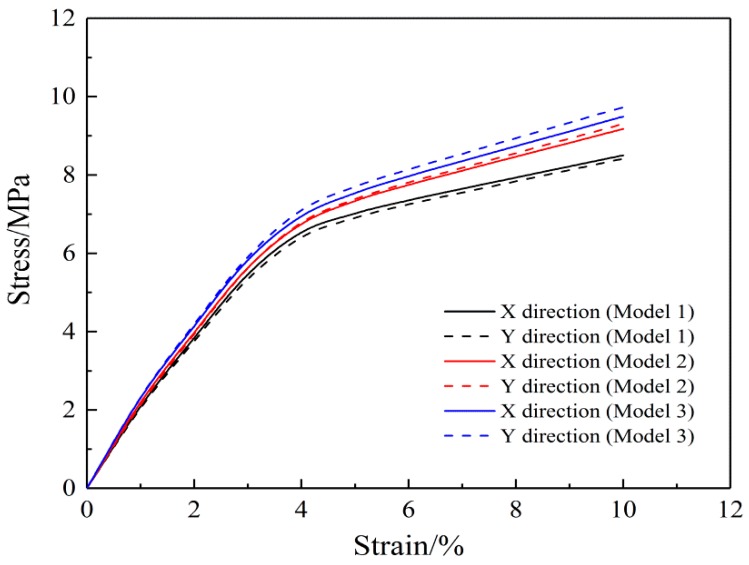
Biaxial tension stress-strain curves of three random models.

The stress-strain curves of the three random models are shown in Figure 8, which show that randomness has no effect on trend of the stress-strain curve. The stress-strain results of FE analysis of the fibrous mats in Model 2 and Model 3 show similar trends as those in Model 1. This indicates that the simulation results are not affected by the randomness of the modeling.

#### 5.1.4. Parameter Analysis

Different structural parameters in the nanofibrous mat model will affect the biaxial tensile FE analysis performance. Thus, the effects of the aspect ratio and porosity of the nanofibrous mats on the biaxial tensile properties of the nanofibrous mats are analyzed.

Fibrous mat models with aspect ratios of 1:1, 2:1, 4:1, and 8:1 were built, and biaxial tensile simulation tests were performed on the models. The constraint widths on both sides were 5%, and the tensile strain on both axes was 10% during biaxial tension. All models maintained a porosity of 75% and were established from 1000 fibers. The element number scale was kept the same.

**Figure 9:**
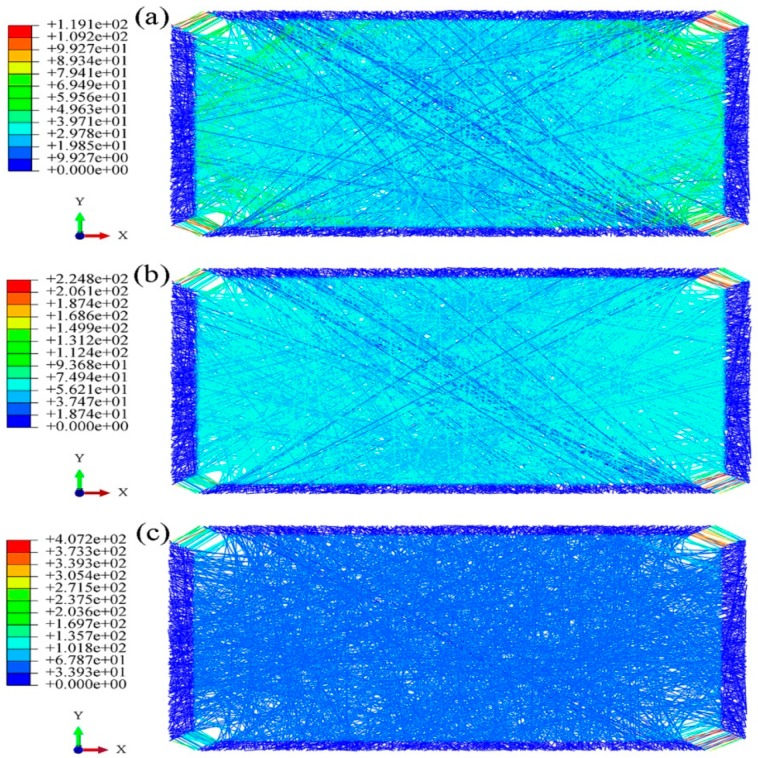
Biaxial tension stress cloud charts (aspect ratio 2:1, porosity 0.75). (**a**) strain = 2%; (**b**) strain = 5%; (**c**) strain = 10%.

**Figure 10:**
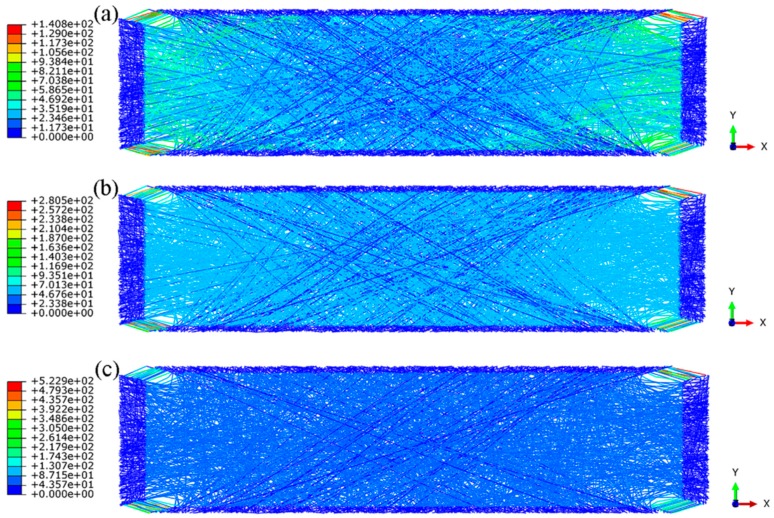
Biaxial tension stress cloud charts (aspect ratio 4:1, porosity 0.75). (**a**) strain = 2%; (**b**) strain = 5%; (**c**) strain = 10%.

**Figure 11:**
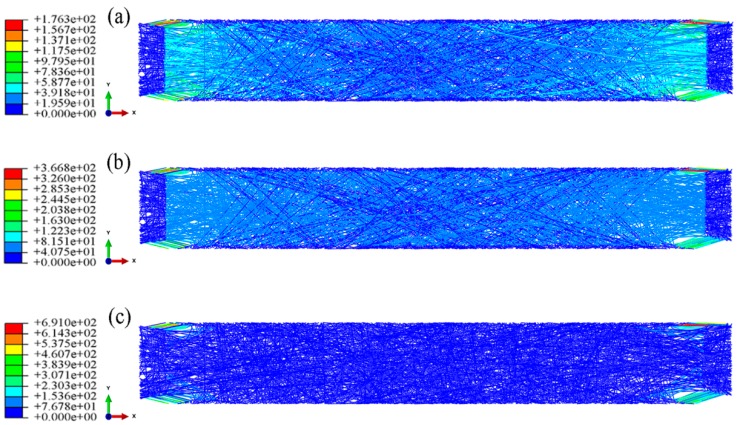
Biaxial tension stress cloud charts (aspect ratio 8:1, porosity 0.75). (**a**) strain = 2%; (**b**) strain = 5%; (**c**) strain = 10%.

Biaxial tensile stress cloud diagrams of the fibrous webs with different aspect ratios are shown in Figure 9, Figure 10 and Figure 11. The fibrous deformation and stress change during biaxial tension are similar to those in the square fibrous mats. First, the fibrous stress in the torn parts of the four corners increases rapidly, causing the fibers to enter the yield phase. After further loading, the fibers in the yield phase gradually increase and diffuse into the interior of the fibrous mats. Finally, almost all fibers enter the yield phase with continuous loading.

**Figure 12:**
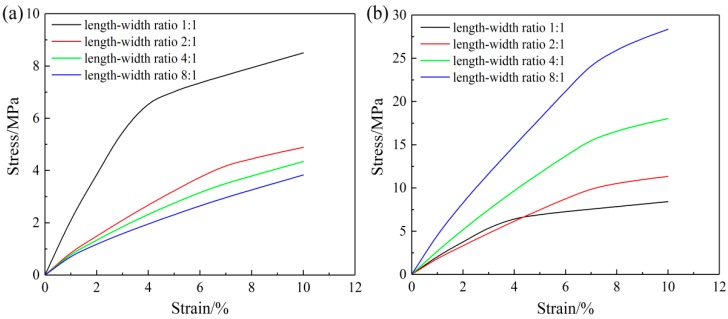
Biaxial tension simulation stress-strain curves of models with four different aspect ratios in the (**a**) X direction and (**b**) Y direction.

A comparison of the biaxial tensile stress-strain curves of the nanofibrous mats with different aspect ratios is shown in Figure 12. When the aspect ratio was 1:1, the stress-strain curves in the X and Y directions are similar. As the aspect ratio increased, the X-direction modulus decreased continuously, while the Y-direction modulus increased continuously. The difference in the modulus in each direction became increasingly significant.

In addition to the aspect ratio, the porosity can also affect the density of the fibrous mats and therefore influence the biaxial tensile test result. FE models of nanofibrous mats with porosities of 60%, 70%, 80%, and 90% were established, and biaxial tensile simulation tests were conducted. During biaxial tension, the constraint width on both sides was 5% with a tensile strain of 10%. All models maintained the same aspect ratio of 1:1, and 1000 fibers were included in the model. A similar element number scale was maintained.

**Figure 13:**
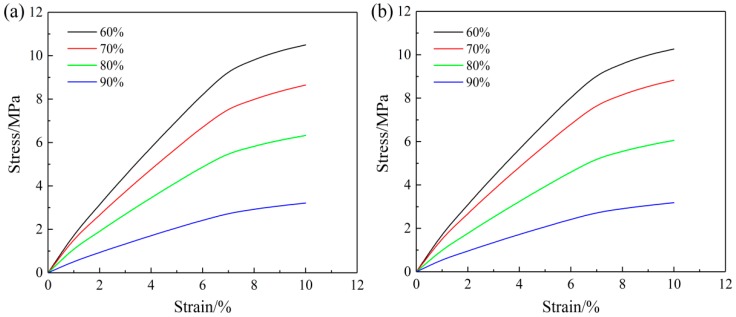
Biaxial tension simulation stress-strain curves of models with four different porosity ratios in the (**a**) X direction and (**b**) Y direction.

A comparison of various porosity stress-strain curves is shown in Figure 13. As the porosity increases, the volume fraction of the fibrous decreases, and the initial modulus in the two directions of the nanofibrous mats also gradually decreases. The change in the trend of the tension curve in the X and Y directions are approximately the same.

### 5.2. Non-uniform Nanofiber Distribution

#### 5.2.1. Finite Element Model

The fibers in the SF/PCL nanofibrous mats formed at a roller speed of 11.88 m∙s^−1^ had a normal distribution of orientations. In addition, numerous fibers are centralized along the Y direction, while few are centralized along the X direction. Based on the test data, a FE model was built to analyze the biaxial tensile response of the orientation distribution of the nanofibrous mats.

The percentage of orientations of the fibers in the nanofibrous mats in Table 1 can be obtained according to Figure 2b. The MATLAB programming and rand function were used for the automatic generation of the fibrous web structure of the probability distribution in the table. 

The methods for random number generation are as follows:

1. The rand(1) function generates a 0–1 random number, prob is a randomly generated 0–1 random number, and the probability of being generated in the 0–1 range is the same;
2. The 0–1 range is divided into 12 sectors according to Table 1, and the probability in the 12 sectors is the probability of each fibrous angle;
3. When the random number prob falls in a certain zone, the corresponding fibrous angle of the zone is generated. For example, if prob = 0.5 falls into the (prob > 0.2993 && prob ≤ 0.7898) zone, then θ = π*(rand(1)*0.0833 + 0.0833*5), where θ is randomly generated in the 0.4165π–0.4998π (75°–90°) zone.

**Figure 14:**
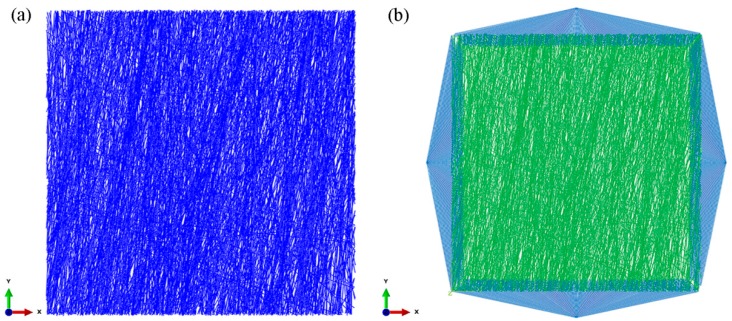
Oriented SF/PCL nanofibrous mats. (**a**) FE model; (**b**) biaxial loading mode.

The FE model of the nanofibrous mats generated by the probability of the fiber orientation distribution is shown in Figure 14a. The model length is 100 μm, the width is 100 μm, the element number is 94940, and the porosity is 75%. The probability of the fiber orientation distribution in the model is in line with the test. In addition, a large number of fibrous are centralized in the Y direction, and very few fibers in the X direction. Biaxial loading of the oriented SF/PCL nanofibrous mats is shown in Figure 14b.

#### 5.2.2. Biaxial Tension Analysis

**Figure 15:**
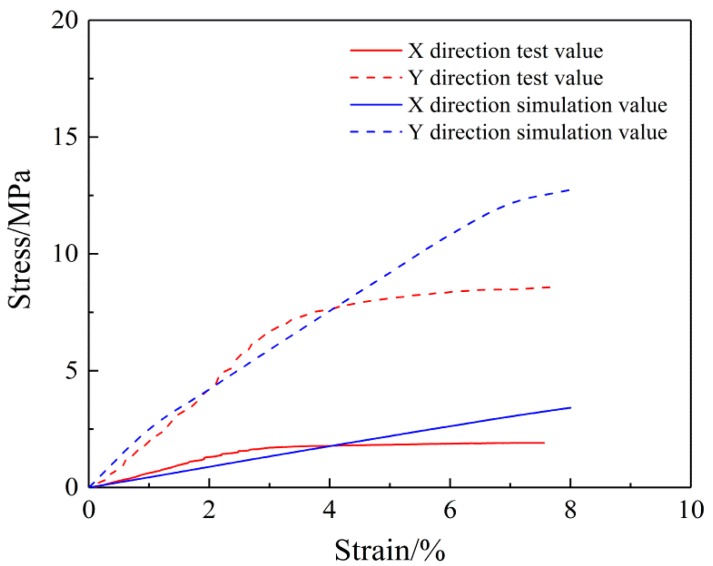
Biaxial stress-strain curves of the oriented nanofibrous mats.

Biaxial tension simulation curves and experimental curves of the oriented SF/PCL nanofibrous mats are shown in Figure 15. This shows the oriented nanofibrous mats and the uniform nanofibrous mats have the same biaxial tensile stress-strain curve. With the tension of the mats, the modulus in both the X and Y direction gradually decreases. However, due to the uneven distribution of fibers, the modulus in the X direction is significantly lower than that in the Y direction. The biaxial tension simulation results are consistent with the test results under small strain. Under large strain, the tension model does not show destruction of the fibers, which contrasts with the test results.

**Figure 16:**
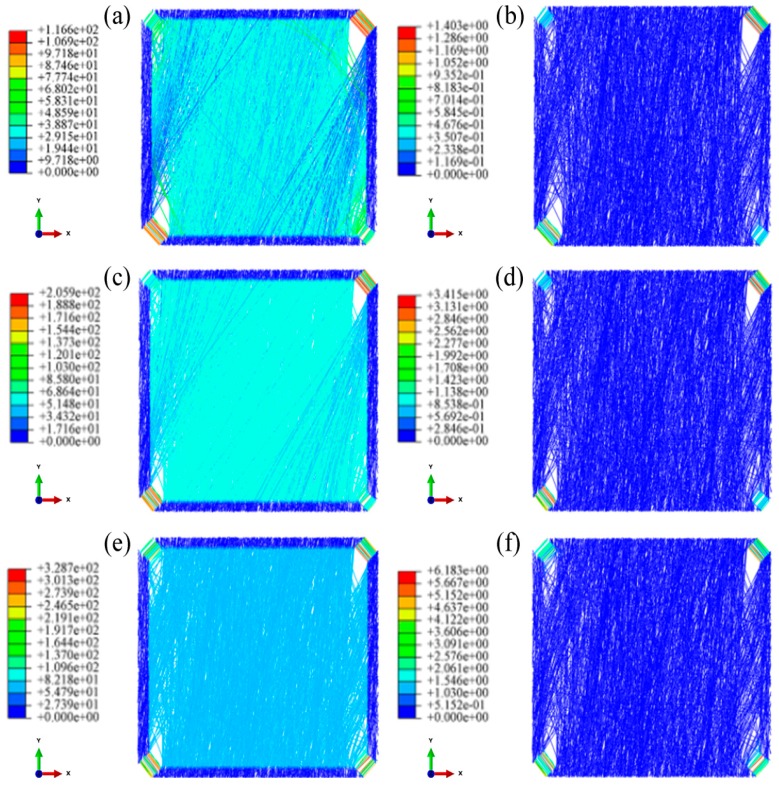
Biaxial tension cloud charts of the oriented nanofibrous mats (aspect ratio 1:1, porosity 0.75). 1. strain = 2%, (**a**) stress (**b**) strain; 2. strain = 5%, (**c**) stress (**d**) strain; 3. strain = 10%, (**e**) stress (**f**) strain.

The biaxial stress change in the oriented nanofibrous mats is shown in Figure 16, which indicates that with biaxial loading, the stress on the fibers rapidly increases, tearing the four corners, followed by the fibers entering the yield phase. With further loading, the number of fibers in the yield phase gradually increases, and the fibers diffuse inside of the fiber membrane. At the end of the test, almost all the fibers entered the yield phase with continuous loading. This shares some similarities with the fiber deformation and stress variation of the uniform nanofibrous mats.

## 6. Conclusions

In this paper, the mechanical response of electrospun SF/PCL nanofibrous mats under biaxial tension was examined using an FE model. This direct microstructure-based numerical approach maintains the relation between the microstructure of the electrospun nanofibrous mats and their deformation behavior. The developed model reproduced the deformation of the nanofibrous mats and the gradual damage mechanism. By analyzing the FE models of several SF/PCL nanofibrous mats, the following conclusions are obtained:

(1) The FE models of the SF/PCL nanofibrous mats (uniform fiber distribution and oriented fiber distribution) were built, and the model membranes were analyzed under biaxial tension to obtain stress-strain curves, which were similar to the test results.
(2) The mats with a random distribution of electrospun nanofibers presented the same mechanical response in both biaxial tension directions. This confirmed the preliminary theoretical formula of biaxial tension. In addition, multiple sets of stochastic models were built, and the FE analysis showed that the simulation results did not depend on the randomness of modeling.
(3) With an increase in the aspect ratio, the modulus of the nanofibrous mats decreases due to the upward tension along the long direction of the fibrous mats. In contrast, the modulus increases due to upward tension of the fibrous mats along the short direction of the membranes. The porosity has a negative effect on the modulus of the nanofibrous mats;
(4) FE models of oriented nanofibrous mats were established. The orientation distribution of the fibers shows clear anisotropy in their mechanical tensile properties. The simulation results are consistent with the experimental results.

In this paper, the biaxial tensile elastic-plastic properties of the nanofibrous mats were obtained. Meanwhile, a set of nanofibrous mats based on the real nanofibrous orientation probability distribution was established for biaxial FE simulation analysis to clarify the relationship between the microstructure and the mechanical properties of electrospun nanofibrous mats. Areas worthy of future in-depth study include the elastic-plastic properties of non-uniformly distributed nanofibrous mats in all directions and the influence of inter-fiber nodes in the microstructure of the electrospun nanofibrous mats on the mechanical properties of the mats.

## Figures and Tables

**Table 1:** Probability distribution of the orientation SF/PCL nanofibrous mats.

Section	Angle (°)	Percentage (%)	Section	Angle (°)	Percentage (%)
1	15	0.27	7	105	17.13
2	30	0.25	8	120	1.17
3	45	1.46	9	135	1.04
4	60	1.45	10	150	0.37
5	75	26.5	11	165	1.23
6	90	49.05	12	180	0.08
